# Morpholinyl silicon phthalocyanine nanoparticles with lysosome cell death and two-photon imaging functions for *in vitro* photodynamic therapy of cancer cells

**DOI:** 10.3389/fbioe.2023.1181448

**Published:** 2023-05-05

**Authors:** Hongjie Yu, Jianling Chen, Xiuqin Chen, Tiantian Zhang, Yuyang Li, Kuizhi Chen, Yiru Peng, Linying Chen

**Affiliations:** ^1^ College of Chemistry and Chemical Engineering, Fujian Provincial Key Laboratory of Advanced Materials Oriented Chemical Engineering, Fujian Province Key Laboratory of Polymer Materials, Fujian Normal University, Fuzhou, China; ^2^ College of Photonic & Electronic Engineering, Fujian Normal University, Fuzhou, China; ^3^ Department of Pathology, The First Affiliated Hospital of Fujian Medical University, Fuzhou, Fujian, China

**Keywords:** morpholine, phthalocyanine, lysosome target, two-photon bio-imaging, photodynamic therapy

## Abstract

The lysosome is an important target for realizing antitumor therapy. Lysosomal cell death exerts significant therapeutic effects on apoptosis and drug-resistance. The development of lysosome-targeting nanoparticles to obtain efficient cancer treatment is challenging. In this article, nanoparticles composed of DSPE@M-SiPc and possessing bright two-photon fluorescence, lysosome targeting ability, and photodynamic therapy multifunctionalities are prepared by encapsulating morpholinyl-substituted silicon phthalocyanine (M-SiPc) with 1,2-distearoyl-sn-glycero-3-phosphoethanolamine-N-[methoxy(poly(ethylene glycol))-2000] (DSPE). Two photon fluorescence bioimaging showed that M-SiPc and DSPE@M-SiPc mainly locate in lysosomes after cellular internalization. Upon irradiation, DSPE@M-SiPc effectively generates reactive oxygen species and damages the function of lysosome, subsequently leading to lysosomal cell death. DSPE@M-SiPc is a promising photosensitizer for cancer treatment.

## 1 Introduction

Photodynamic therapy (PDT) is a non-invasive treatment that involves photosensitizers (PSs), specific wavelength light, and oxygen to generate highly toxic reactive oxygen species (ROS), leading to cancer cell death (Ali et al., 2022; Biyiklioglu et al., 2022). Some traditional methods, such as chemotherapy, radiotherapy, and surgical treatment, are applied in cancer treatment. Compared with non-invasive therapy, traditional therapy methods not only kill cancer cells/tumors, but also damage normal cells, which generates obviously side effects (Narayanamurthy et al., 2018). PDT is a highly safe, low-invasive, low dark toxicity, controllable, and high cure treatment method with minimal side effects (Xu et al., 2023). The design and synthesis of novel and effective PSs is appealing (Wu et al., 2021; Wang et al., 2022b; Wu et al., 2022; Zhan et al., 2022; Wu et al., 2023a; Wu et al., 2023b). In addition, PSs as fluorescence imaging agents have attracted great interest because they can visualize living cells, tumor tissues, and even the entire body with rapid feedback and high resolution and sensitivity (Jiang et al., 2021). In particular, two-photon fluorescence bioimaging exhibits some merits such as spatial selectivity, deep tissue penetration, high spatial resolution, and low photodamage, and has been utilized for imaging of tissue/cells (Liu et al., 2022). However, most conventional PSs generally suffer from aggregation of their rigid structures or hydrophobic features, leading to poor emission in biological imaging and limited PDT efficiency (Cheng et al., 2022). Therefore, the fabrication of multifunctional PSs with both bright fluorescence and high PDT efficiency is needed.

Phthalocyanines (Pcs) and their metal derivatives are promising second generation phthalocyanines. Pcs have received considerable attention due to their strong absorption, high ROS quantum yield, and low dark toxicity in the light therapy window (Yüzeroğlu et al., 2021). However, Pcs also suffer from numerous disadvantageous including low water solubility, serious aggregation tendencies under physiological conditions, and poor cancer cell selectivity, leading to insufficient PDT efficiency (Ou et al., 2021). At the same time, the short lifetime (<40 ns) and diffusion radius (<20 nm) of ROS produced by Pcs greatly limit their photodynamic action range and reduce their treatment efficacies (Chen et al., 2022). In order to overcome these shortcomings, many organelles targeting PSs have been synthesized and applied in PDT (Wang et al., 2022b; Ding et al., 2022; Li et al., 2022; Luo et al., 2022).

Lysosomes, acidic, membrane-bound organelles, play an important role in cell apoptosis, immune defense, intracellular digestion, and special secretory functions (Bohnert and Johnson, 2022). Lysosome cell death has been considered a promising antitumor strategy. One approach is to disrupt the lysosomal activity by inducing partial lysosomal membrane permeabilization through ROS, ultimately resulting in lysosomal cell death (Nene et al., 2019). Importantly, lysosomal cell death avoids the classic caspase-dependent apoptosis pathway, thereby establishing a special method for targeting apoptosis and drug-resistant cancer. Meanwhile, lysosomes constantly change their morphology and spatial distribution due to their highly dynamic nature (Barral et al., 2022). Therefore, non-invasive, accurate imaging lysosomes will help in monitoring the PSs distribution and in understanding the lysosomal cell death mechanism.

Morpholine and its substituted derivatives are widely employed pharmaceuticals and are biologically active molecules because of their special physicochemical, biological, and metabolic properties (Dwivedi et al., 2022; Wangngae et al., 2022). Moreover, since the morpholinyl moiety has a pKa of 5–6 and can be protonated in an acidic environment (pH 4.5–5.0), it is a suitable lysosome-targeting unit (Jiang et al., 2017). The introduction of morpholinyl moieties to axial/periphery positions on silicon phthalocyanine has been demonstrated to modulate its physicochemical properties. For example, 1-morpholinopropan-2-ol/3-morpholinophenol 2-morpholinyl ethoxy–substituted silicon phthalocyanines have been demonstrated to improve the photophysical properties of silicon phthalocyanines and enhance their PDT efficacies (Zhu et al., 2006; Zhang and Wang, 2011; Wang et al., 2022a). However, the fate of these lysosomal-targeted silicon phthalocyanines in cancer cells has not been reported.

In this study, a novel morpholinyl-substituted silicon phthalocyanine, named di-(4′-(2-(morpholinyl-ethoxycarbonyl-1,1′-biphenyl-4-ethoxy))) axially substituted silicon phthalocyanine (M-SiPc), and its water-soluble polymeric nanoparticle (DSPE@M-SiPc) were prepared. DSPE@M-SiPc was prepared by encapsulating M-SiPc with 2-distearoyl-sn-glycero-3-phosphoethanolamine-N-[methoxy(poly(ethylene glycol))-2000] (DSPE). DSPE@M-SiPc exhibited NIR emission, two-photon fluorescence bioimaging, and lysosome targeting multifunctionalities. *In vitro* PDT studies revealed that DSPE@M-SiPc performed well in imaging-guided photodynamic therapy of tumors.

## 2 Experimental section

### 2.1 Materials

All chemicals were analytical reagent grade quality and were obtained from commercial suppliers. 4-carboxyphenylboric acid, 4-dimethyl aminopyridine (DMAP), 1-ethyl-(3-dimethyl aminopropyl) carboimide hydrochloride (EDC·HCl), 2-morpholine ethanol, tetra triphenylphosphine palladium, and 4-bromobenzyl alcohol were purchased from SANN Chemical Technology Co. (Shanghai, China). The common chemical reagents dimethyl sulfoxide (DMSO), dichloromethane, silica gel (100–200 mesh), acetone, and methanol were purchased from Sino-pharm Chemical Reagent Co. (Shanghai, China) for direct use. 1,2-distearoyl-sn-glycero-3-phosphoethanolamine-N-[methoxy(poly(ethylene glycol))-2000] (DSPE-mPEG_2000_) was purchased from Shanghai Yayi Biotechnology Co. (Shanghai, China). Lysosomal dye (lysotracker DND-99), annexin V-FITC/PI, dulbecco’s modified eagle medium (DMEM), high glucose medium, trypsin, fetal bovine serum (FBS), and PBS buffer were purchased from Hyclone, United States. 3-(4,5-dimethylthiazole-2)-2,5-diphenyltetrazolium bromide (MTT) was purchased from Sigma Aldrich (Shanghai, China). Breast cancer cells (MCF-7) were purchased from Shanghai Ke Feng Biotechnology Co. (Shanghai, China).

### 2.2 Synthesis of 4-(4-(2-morpholine ethoxy) carbonyl) phenylboronic acid (M-pha)

A mixture of 4-dimethyl aminopyridine (DMAP) (0.60 mmol, 0.0700 g), 1-ethyl-(3-dimethyl aminopropyl) carboimide hydrochloride (EDC·HCl) (2.50 mmol, 0.5000 g), and tetracarboxyphenylboric acid (2.00 mmol, 0.3300 g) was added to dichloromethane (20.00 mL). After stirring for 30 min, 2-morpholine ethanol (4.00 mmol, 0.5300 g) was added dropwise and the mixture was stirred at room temperature for 48 h. The mixture was extracted with dichloromethane (50.00 mL) three times. The organic phase was dried with anhydrous MgSO_4_ and filtered. The organic phase was collected and after the organic solvent was removed a white crude product was obtained. The crude product was purified twice with dichloromethane/methanol (v/v = 10/1) as the eluent using a silica gel column as the solid phase. After drying, a white solid was obtained with a yield of 48.00%. FT-IR ν_max/cm_^−1^: 3344 (aromatic C–H), 3046–2978 (aliphatic C–H), 1697, 1659, 1414 (C=C), 1347, 1268 (aromatic–O–aromatic), 1098, 1019, 704. ^1^HNMR (400 Hz, CH_3_OD): *δ* = 8.02 (d, J = 8 Hz, 2H), 7.76 (s, 2H), 4.50 (m, 2H), 3.73 (m, 4H), 2.85 (m, 2H), 2.65 (m, 4H). ESI-MS for C_13_H_18_NO_5_B (m/z): 278.13, found: 301.12 [M + Na]^+^.

### 2.3 Synthesis of 4′-hydroxymethyl-(1,1′-biphenyl)-4-formic acid-2-(2-morpholinyl) ethyl ester (M-OH)

A mixture of M-pha (2.00 mmol, 0.5900 g), tetrakis (triphenylphosphine) palladium (0.06 mmol, 0.0800 g), 4-bromobenzyl alcohol (1.50 mmol, 0.3000 g), potassium carbonate (2.00 mol/L, 15.00 mL), and tetrahydrofuran (30.00 mL) was refluxed under nitrogen for 12 h. The organic layer was dried with anhydrous MgSO_4_ and filtered. The organic layer was collected and after the organic solvent was removed a white crude product was obtained. The crude product was purified twice with dichloromethane/methanol (v/v = 50/1) as the eluent in silica gel column chromatography. A white powder was obtained with a yield of 20.00%. FT-IR ν_max/cm_^−1^: 3202 (aromatic C–H), 2862–2825 (aliphatic C–H), 1705 (C=O), 1607, 1458 (C=C), 1272, 1107 (aromatic–O–aromatic), 939, 828, 767. ^1^H NMR (400 MHz, CDCl_3_): 8.12 (d, J = 8 Hz, 2H), 7.70 (d, J = 8 Hz, 2H), 7.65 (d, J = 8 Hz, 2H), 7.50 (d, J = 8 Hz, 2H), 4.80 (s, 2H), 4.67 (m, 2H), 4.52 (m, 2H), 3.77 (m, 4H), 2.84 (m, 2H), 2.63 (m, 4H). ESI-MS for C_20_H_23_NO_4_ (m/z): 341.16, found: 341.16 [M]^+^.

### 2.4 Synthesis of di-(4′-(2-(morpholinyl-ethoxycarbonyl-1,1′-biphenyl-4-ethoxy)) substituted silicon phthalocyanine (M-SiPc)

A mixture of M-OH (1.00 mmol, 0.3300 g), dichlorosilicon phthalocyanine (0.33 mmol, 0.2100 g), sodium hydride (1.30 mmol, 0.0330 g), and toluene (30.00 mL) was refluxed under nitrogen for 48 h. After the mixture cooled to room temperature, the crude product was purified three times by silica gel column chromatography with dichloromethane/acetone (v/v = 6/1) as the eluent. A blue solid was obtained with a yield of 30.00%. FT-IR ν_max/cm_^−1^: 3057 (aromatic C–H), 2960–2842 (aliphatic C–H), 1710, 1612, 1433 (C=C), 1333, 1272 (aromatic–O–aromatic), 1081 (Si–O), 735. ^1^H NMR (400 MHz, CDCl_3_): *δ* = 9.59 (m, 8H), 8.32 (m, 8H), 7.91 (d, J = 8 Hz, 4H), 7.15 (d, J = 8 Hz, 4H), 6.38 (d, J = 8 Hz, 4H), 4.41 (m, 4H), 4.39 (m, 4H), 3.74 (m, 8H), 2.62 (m, 4H), 2.60 (m, 8H), −0.64 (d, J = 8 Hz, 4H). ESI-MS for C_72_H_64_N_10_O_8_Si (m/z): 1220.44, found: 1220.45 [M]^+^.

### 2.5 Synthesis of DSPE@M-SiPc nanoparticles

**SCHEME 1:**
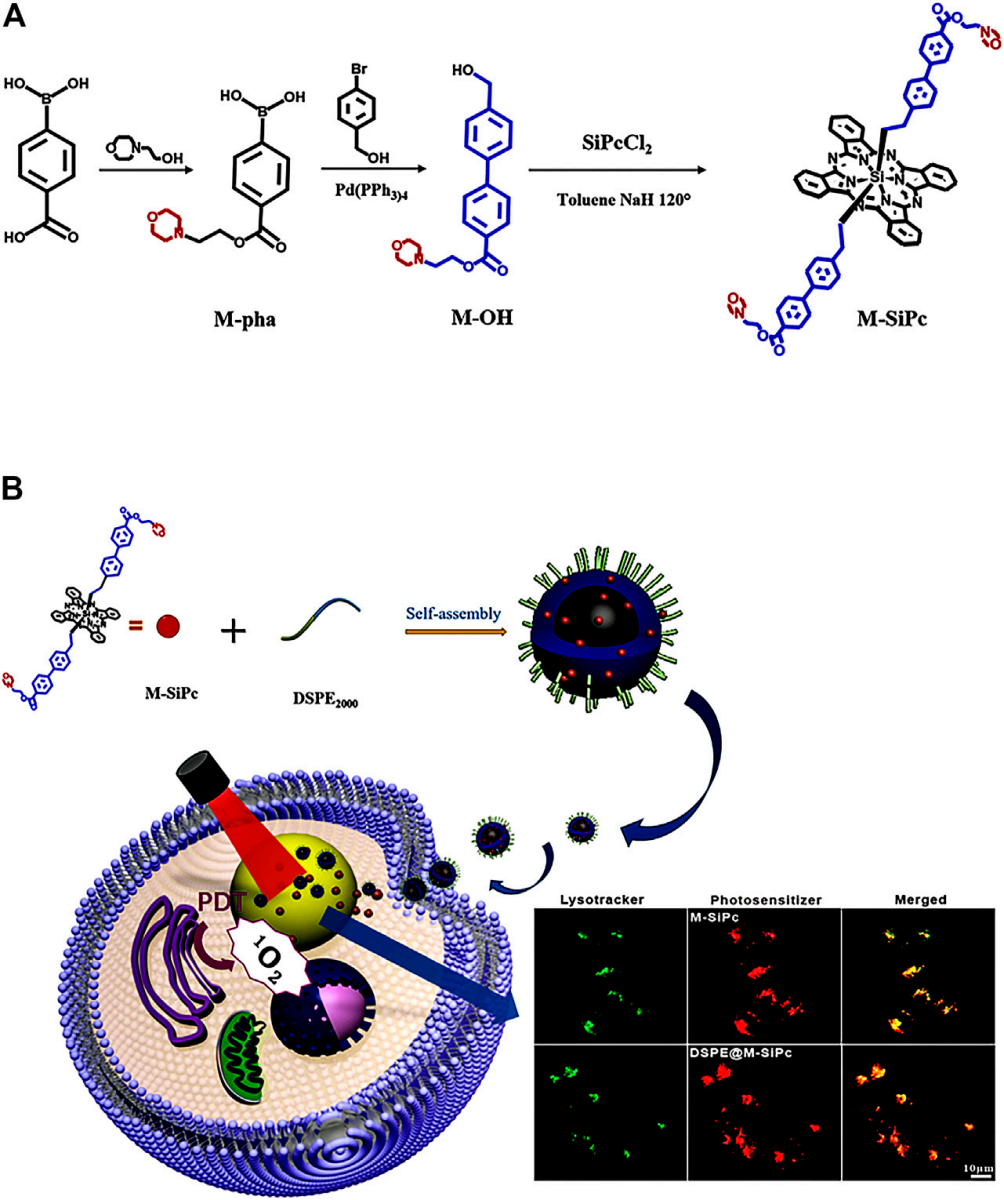
**(A)** Scheme for the synthesis of morpholinyl-substituted silicon phthalocyanine. **(B)** Preparation and intracellular distributions of DSPE@M-SiPc nanoparticles in MCF-7 cells.

The amphiphilic block copolymer 1,2-distearoyl-sn-glycero-3-phosphoethanolamine-N-[methoxy(poly(ethylene glycol))-2000] (DSPE-PEG_2000_) (1.00 mg) was dissolved in an M-SiPc solution (1 × 10^−4^ mol/L, 1.00 mL) and sonicated for 2 min. Subsequently, deionized water (9.00 mL) was added to the mixture which was stirred vigorously at room temperature for 24 h. The aqueous suspension was further purified using a 0.22 μm membrane filter. The filtrate was collected, and the obtained product was stored in the dark at 2–8°C. The flowchart as shown in Scheme 1B.

### 2.6 Two-photon fluorescence imaging of M-SiPc and DSPE@M-SiPc in MCF-7 breast cancer cells

Cell culture and cytotoxicity evaluation: MCF-7 cells were cultured in a cell culture medium at 37°C and 5% CO_2_. The cytotoxicity of DSPE@M-SiPc was determined using the 3-(4,5-dimethylthiazol-2-yl)-2,5-diphenyltetrazolium bromide (MTT) method. The cells were co-incubated with different concentrations of DSPE@M-SiPc (0, 1, 2, 3, and 4 μM) for 24 h. Subsequently, MTT (20 μL, 5 mg/mL) was added and further incubated with MCF-7 cells for 4 h.

The relative cell viability was calculated using Eq. 1:

$$Relative\;cell\;viablity\left(\%\right)=\frac{{0D}_{sample}-0D_{blank}}{0D_{control}-0D_{blank}}\times 100\%$$ (1)

Subcellular localization: MCF-7 cells were cultured in a 35 mm confocal culture dish for 24 h, and then M-SiPc (4 μM) and DSPE@M-SiPc (4 μM) were co-cultured with cells for 12 h. Subsequently, lysotracker DND-99 (0.17 mg/mL, 150 μL) was added and co-incubated with cells for a further 30 min at 4°C. DSPE@M-SiPc was excited at 830 nm using a femtosecond laser and their fluorescence was monitored at 650–700 nm. Lysotracker DND-99 was excited using a 488 nm laser and its fluorescence was monitored at 600–650 nm.

Intracellular ROS generation ability: CM-H_2_DCFDA (5 μM) was used as a probe to determine the intracellular ROS production ability. MCF-7 cells were cultured in a 35 mm glass plate for 24 h at a density per plate of 1.0 × 10^4^ cells. Subsequently, DSPE@M-SiPc (4 μM) was added and co-incubated for 12 h. The cells were irradiated with a 671 nm laser (40 mW/cm^2^, 10 min) and a control experiment was conducted without irradiation. Subsequently, the cells were mixed with CM-H_2_DCFDA (5 μM) and incubated in 0.5 mL fresh medium for 20 min. Finally, the culture medium was aspirated, and the cells were washed with PBS three times. ROS generation ability for DSPE@M-SiPc was observed by confocal laser scanning microscopy (CLSM).

*In vitro* photodynamic therapy efficiency: The photodynamic therapy efficacies of DSPE@M-SiPc were determined using the MTT method. Briefly, 100 μL medium mixed with DSPE@M-SiPc (0, 1, 2, 3, and 4 μM) was added into the MCF-7 cells, and the cells were incubated for 24 h. Subsequently, the cells were irradiated with a laser (671 nm, 100 mW/cm^2^) for 10 min, Finally, MTT (20 μL, 5 mg/mL) was co-incubated with MCF-7 cells for 4 h.

Annexin V-FITC/PI flow cytometry: MCF-7 cells were seeded overnight in 12-well culture plates at a density of 1 × 10^4^ cells per well. The cells were removed and replaced with fresh DMEM containing DSPE@M-SiPc (C_DSPE@M-SiPc_ = 4 μM) and a blank group was directly cultured in a fresh DMEM medium for 8 h. Subsequently, the cells were irradiated with a 671 nm laser (100 mW/cm^2^) for 10 min and further incubated for another 4 h. The resulting cells were stained with a mixture of annexin V-FITC (5 µL) and propidium iodide (10 µL) for 15 min. The mixture was analyzed using flow cytometry to assess apoptotic cells. All experiments were performed in triplicate.

## 3 Results and discussion

### 3.1 Synthesis and characterization

The synthetic scheme for morpholinyl-substituted silicon phthalocyanines (M-SiPc) is shown in Scheme 1A. Its structure was characterized by ^1^H NMR, FT-IR, and ESI-MS. M-SiPc was soluble in a number of common solvents, such as dichloromethane (CH_2_Cl_2_), dimethylsulfoxide (DMSO), N, N-dimethylformamide (DMF), and tetrahydrofuran (THF). The ^1^H NMR spectrum of M-SiPc was consistent with the proposed structure (Supplementary Figures S2, S5, S8). Two sets of resonances were observed at 9.59 and 8.32 ppm, integrated for eight protons of silicon phthalocyanine. Doublet resonances for aromatic protons of axial substitutions appeared at 7.91, 7.15, and 6.38 ppm. The resonances at 4.39 and 2.62 ppm were assigned to methylene protons. In addition, the ESI-MS spectrum showed that each product has a strong singlet charged molecular ion peak, which conformed to the proposed structure (Supplementary Figures S3, S6, S9).

### 3.2 Photophysical and photochemical properties

**FIGURE 1:**
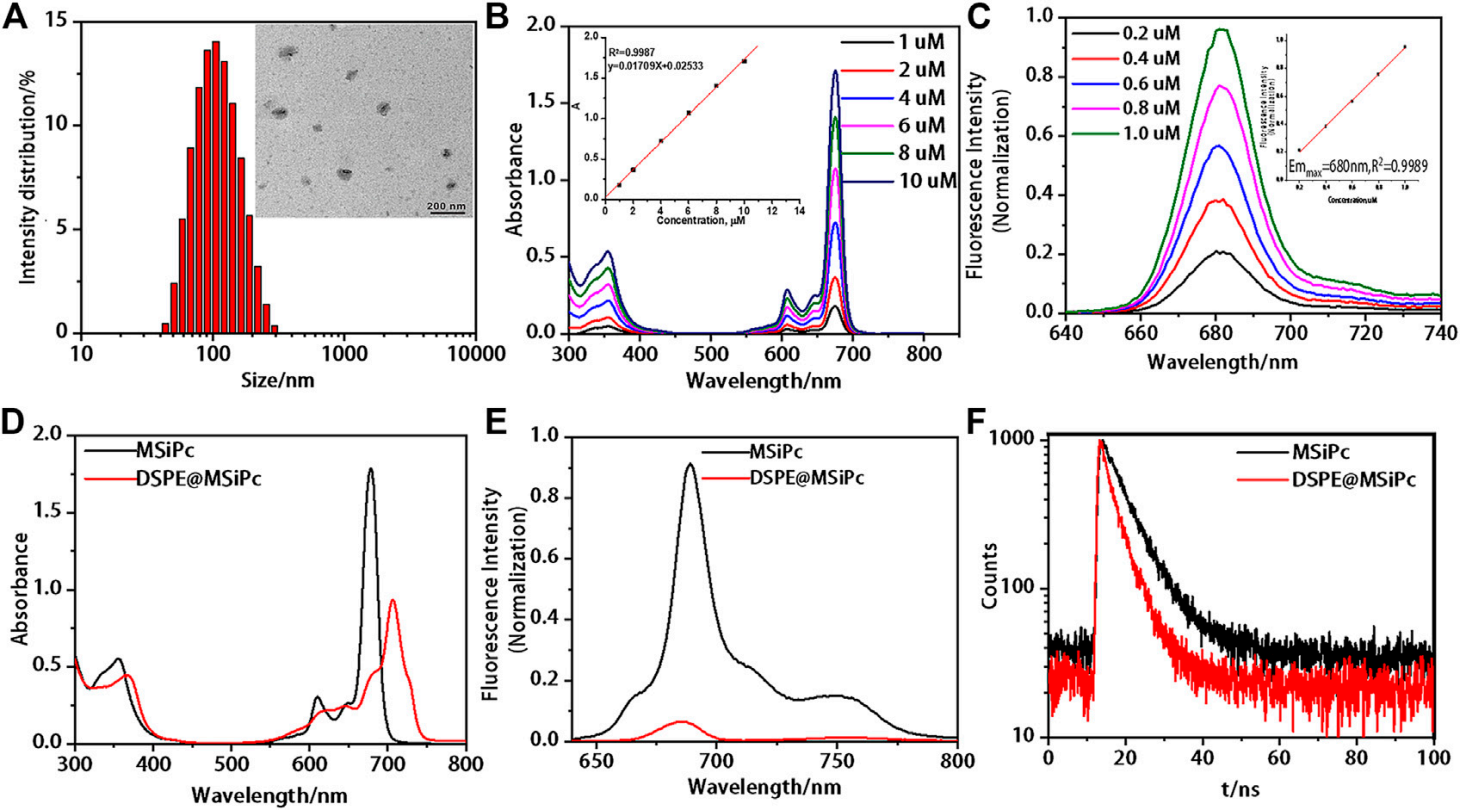
Photophysical properties of M-SiPc and DSPE@M-SiPc. **(A)** TEM image and DLS size of DSPE@ M-SiPc. **(B)** UV-Vis spectra of M-SiPc in DMF (the inset shows the absorbance at 676 nm as a function of concentration). **(C)** Fluorescence spectra of M-SiPc in DMF (the inset shows the fluorescence intensity at 680 nm as a function of concentration, λ_ex_ = 610 nm). **(D)** UV-Vis spectra of M-SiPc in DMSO and DSPE@ M-SiPc in water (C_M-SiPc_ = 10 μM). **(E)** Fluorescence spectra of M-SiPc in DMSO and DSPE@M-SiPc in water (C_M-SiPc_ = 10 μM, λ_ex_ = 610 nm). **(F)** Fluorescence decay curves of DSPE@ M-SiPc and M-SiPc (C_M-SiPc_ = 10 μM, λ_ex_ = 610 nm).

The morphology of DSPE@M-SiPc was investigated by transmission electron microscopy (TEM). DSPE@M-SiPc was spherical with an average diameter of approximately 90 nm and a hydrodynamic dimension of approximately 100 nm, with a high dispersity in water (Figure 1A). The average size of DSPE@M-SiPc observed via TEM was smaller than that observed in water, which could be a result of the swelling of the nanoparticles in water (Chen et al., 2019). The encapsulation efficiency (EE%) of M-SiPc in DSPE@M-SiPc was calculated according to Supplementary Equation S1 and was found to be 50.0%. The drug loading (DL%) was calculated according to Supplementary Equation S2 and was found to be 6.2%.

UV-Vis spectra of M-SiPc in DMF are shown in Figure 1B. A B band at 355 nm and a typical non-aggregated Q band at 676 nm were observed. As the concentration of M-SiPc increased, no significant change was observed in the position of the Q-band. Fluorescence spectra of M-SiPc in DMF are shown in Figure 1C. The maximum emission peak of M-SiPc in DMF was located at 680 nm. There was a good linear relationship between the fluorescence intensity and concentration. The fluorescence quantum yield of M-SiPc was calculated according to Supplementary Equation S3 and was found to be 0.37. The singlet oxygen generating ability of M-SiPc was evaluated by a UV-Vis spectroscopic method, using 1,3-diphenylisobenzofuran (DPBF) as the scavenger (Supplementary Figure S10). The singlet-oxygen quantum yield of M-SiPc was calculated according to Supplementary Equations S4, S5 and was found to be 0.38.

In order to endow water solubility to M-SiPc, it was encapsulated with 1,2-distearoyl-sn-glycero-3-phosphoethanolamine-N-[methoxy(poly(ethylene glycol))-2000] (DSPE) to prepare DSPE@M-SiPc. DSPE@M-SiPc dispersed well in water. The Q band of DSPE@M-SiPc was located at 705 nm, which was 27 nm red-shift compared to that of M-SiPc in DMSO (Figure 1D). The red-shift of the Q band of DSPE@M-SiPc can be attributed to the interaction between M-SiPc and DSPE. Compared with M-SiPc, the fluorescence intensity of DSPE@M-SiPc decreased in water (Figure 1E). The intensity of absorption and fluorescence of DSPE@M-SiPc decreased, which is ascribed to the aggregation of M-SiPc in the nanoparticles (Nyokong, 2007; Pan et al., 2019). The fluorescence lifetimes (*τ*) of DSPE@M-SiPc and M-SiPc were calculated according to Supplementary Equation S6 and were found to be 4.22 and 6.75 ns, respectively (Figure 1F). Compared with M-SiPc, the fluorescence lifetime of DSPE@M-SiPc was shortened. This can be attributed to the aggregation of M-SiPc in the nanoparticle micro-environment (Nyokong, 2007).

### 3.3 Lysosome-targeting capacity of DSPE@M-SiPc and M-SiPc

**FIGURE 2:**
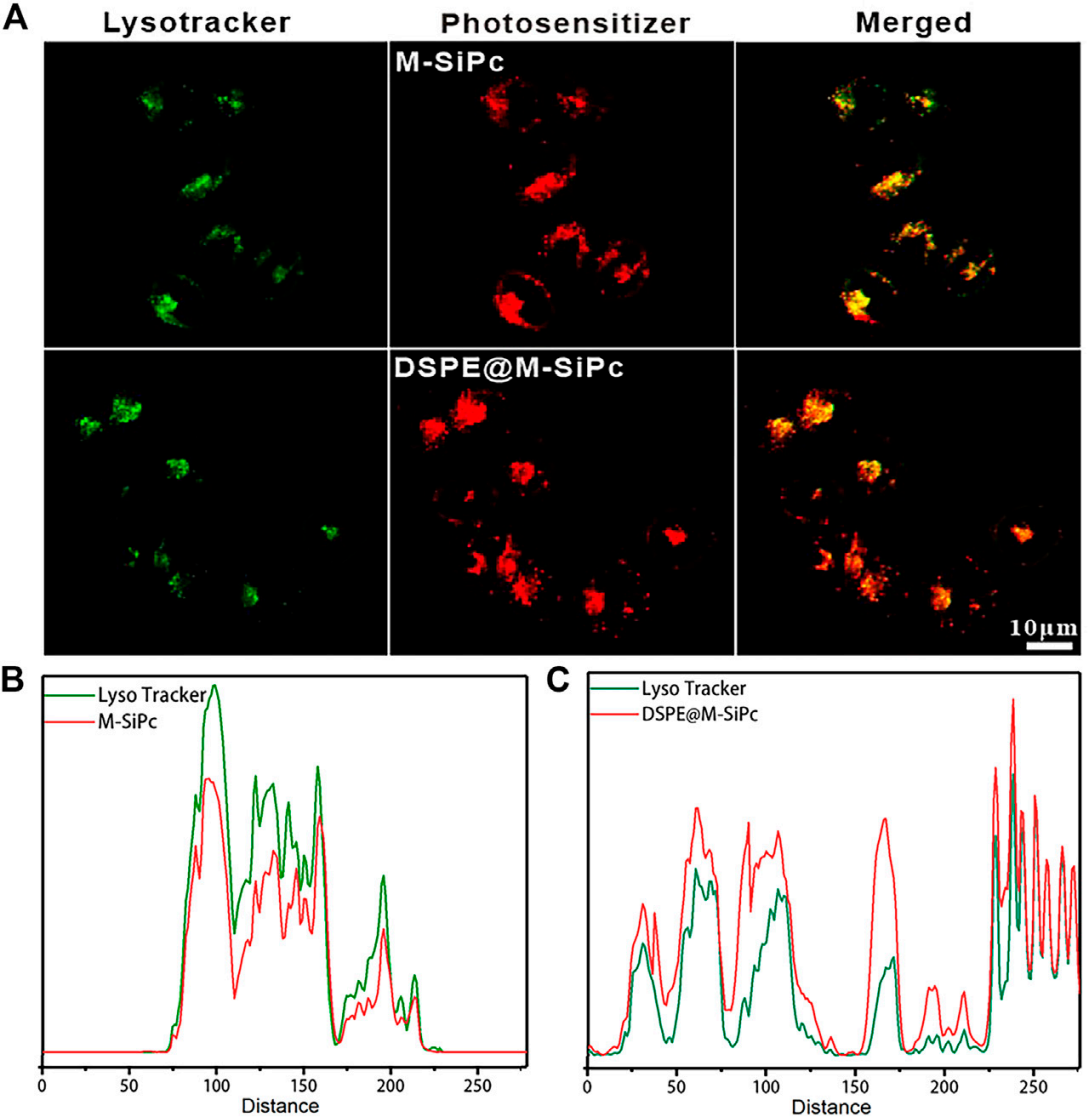
Subcellular localization of M-SiPc and DSPE@M-SiPc in MCF-7 cells observed by CLSM. **(A)** Intracellular distributions of DSPE@M-SiPc, M-SiPc (red, excited at 860 nm by a femtosecond laser and their fluorescence was monitored at 650–700 nm), lysosomes stained by the commercial lysotracker DND-99 (green, excited by a 488 nm laser and their fluorescence was monitored at 510–530 nm), and the merged images of DSPE@M-SiPc, M-SiPc, and lysotracker green. **(B)** Co-localization curve of M-SiPc and lysotracker. **(C)** Co-localization curve of DSPE@M-SiPc and lysotracker (lysotracker was excited by a 488 nm laser and their fluorescence was monitored at 600–650 nm. M-SiPc and DSPE@M-SiPc were excited at 860 nm by a femtosecond laser and their fluorescence was monitored at 650–700 nm).

In our previous study, DSPE was labeled with a green fluorescent probe. FITC were used as nanocarriers to encapsulate cholesterol silicon phthalocyanine (Chol-Pc). The fate of FITC-DSPE@Chol-Pc was determined by two-photon fluorescence imaging. We found that the nanocarrier DSPE retained at the plasma membrane began to be transported into the cytoplasm after the Chol-Pc was released into MCF-7 cells (Chen et al., 2019). According to the above result, we propose that a similar mechanism occurs for DSPE@M-SiPc delivered to lysosome. After DSPE@M-SiPc reached the cell membrane, M-SiPc separated from DSPE-PEG, was transported into the cytoplasm, and targeted lysosome alone. To validate the subcellular organelle-targeting function of DSPE@M-SiPc and M-SiPc, their intracellular distributions were studied using a two-photon laser scanning microscope. The commercial dye lysotracker green was employed for prior labelling of the lysosomes. As shown in Figures 2A‐C, excellent accumulation of DSPE@M-SiPc and M-SiPc in the lysosomes was accomplished, as supported by high-degree co-localizations of the DSPE@M-SiPc and M-SiPc (red) and the commercial lysotracker DND-99 (green). The co-location coefficients were calculated to be approximately 0.88 for M-SiPc and 0.84 for DSPE@M-SiPc. The lysosome targeting ability of DSPE@M-SiPc and M-SiPc can be explained by the fact that the morpholinyl moiety has a pKa of 5–6 and can be protonated within the lysosome acidic environment (pH 4.5–5.0).

### 3.4 *In vitro* photodynamic efficacy of DSPE@M-SiPc against MCF-7 cells

**FIGURE 3:**
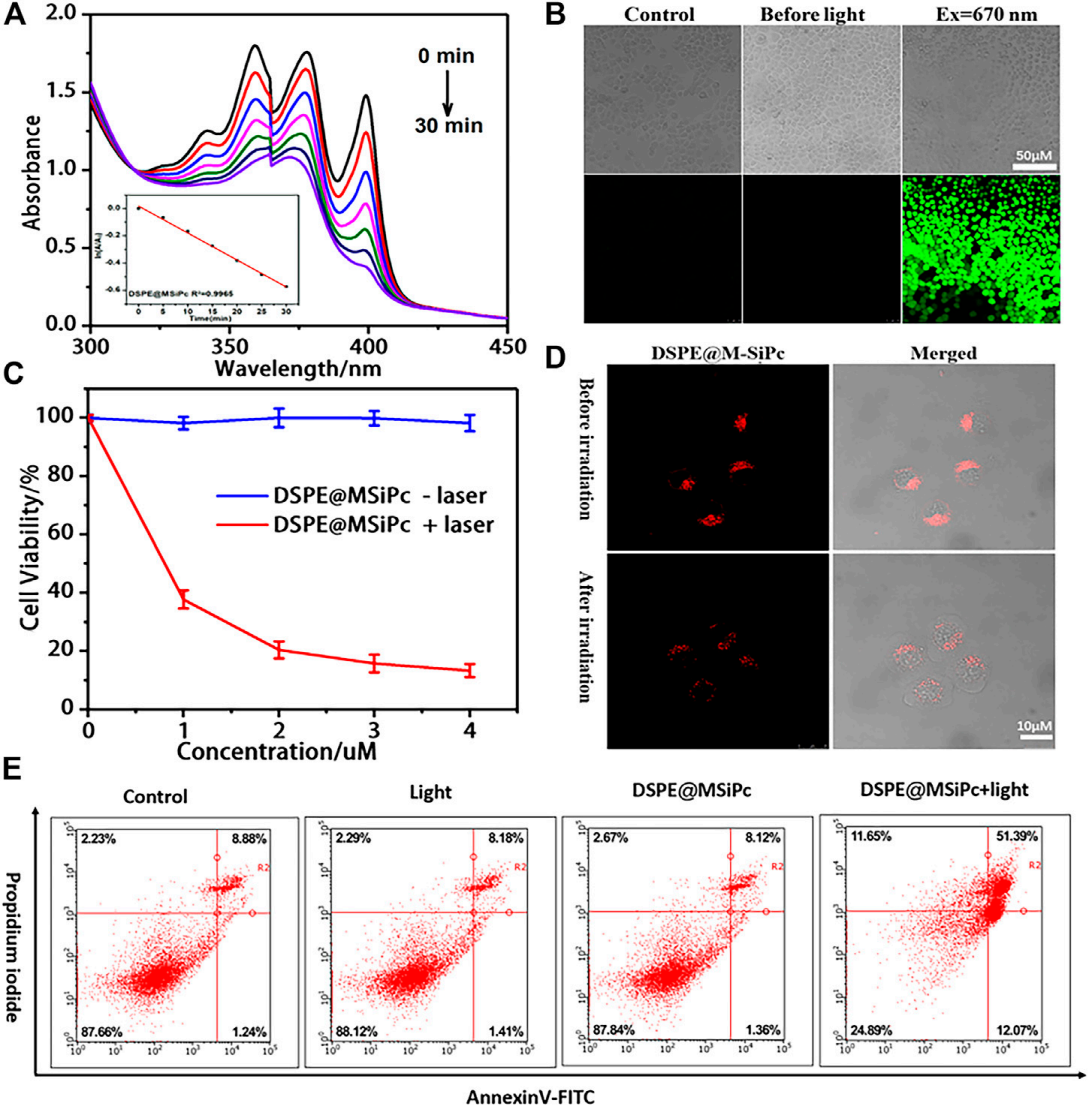
**(A)** UV-vis spectra of ABDA in the presence of DSPE@M-SiPc irradiated by a 671 nm laser (100 mW/cm^2^). **(B)** Intracellular ROS generation ability of DSPE@M-SiPc in MCF-7 cells irradiated by a 671 nm laser (100 mW/cm^2^, 10 min), using H_2_DCFDA as a probe for CLSM. **(C)** Concentration-dependent photocytotoxicity of DSPE@M-SiPc against MCF-7 cells; **(D)** Two photon images of MCF-7 cells incubated with DSPE@M-SiPc (excited at 860 nm by a femtosecond laser and their fluorescence monitored at 650–700 nm), before and after irradiation with a 671 nm laser (100 mW/cm^2^) for 10 min. **(E)** Flow cytometric analysis of cell apoptosis/necrosis after laser treatment.

The ROS generation ability of DSPE@M-SiPc was assessed using ABDA as a probe. Upon irradiation with a 671 nm laser (100 mW/cm^2^), the absorption of the ABDA solution at 378 nm decreased rapidly in the presence of DSPE@M-SiPc, suggesting the efficient generation of ROS (Figure 3A). In order to detect the ROS generation ability in cells, [5-(and-6)-chloromethyl-2′,7′-dichlorodihydrofluorescein diacetate acetyl ester] (CM-H2DCFDA) was used as an indicator. Obvious green fluorescence was observed for cells treated with CM-H_2_DCFDA and DSPE@M-SiPc, confirming valid ROS generation by DSPE@M-SiPc upon irradiation. Moreover, no significant fluorescence was observed in the absence of DSPE@M-SiPc (Figure 3B). The above results indicated that DSPE@M-SiPc can generate ROS in MCF-7 cells.

The cell viability of DSPE@M-SiPc with/without 671 nm laser irradiation was assessed using a CCK-8 assay. DSPE@M-SiPc exhibited almost no cytotoxicity in the absence of light (Figure 3C). This result revealed that DSPE@M-SiPc has biocompatibility in cells. Upon irradiation with a 671 nm laser (100 mW/cm^2^) for 10 min and repeating this experiment three times, the IC_50_ value for DSPE@M-SiPc was found to be 0.77 μM. After incubation with DSPE@M-SiPc (4 μM), and upon irradiation by a 671 nm laser (100 mW/cm^2^) for 10 min, the cell viability decreased to 15%.

To understand the cell death mechanism induced by DSPE@M-SiPc mediated PDT, the annexin V-fluorescein isothiocyanate (FITC) and propidium iodide (PI) co-staining method was applied, and the results were analyzed by flow cytometry (Tam et al., 2022). As shown in Figure 3E, without irradiation, the majority of the MCF-7 cells survived. Only approximately 9% of the cells showed apoptosis, indicating that DSPE@M-SiPc exhibited nontoxicity in the absence of light. Upon irradiation (671 nm, 100 mW/cm^2^, 10 min), the percentage cells that survived decreased to 24%, while the percentage of apoptotic cells increased to 68%. As the percentage of necrotic cells was negligible, this result strongly indicated that apoptosis is the major cell death pathway in the photodynamic action of DSPE@M-SiPc.

Were the organizations of lysosomes in cells destroyed after laser irradiation? To answer this question, the morphological changes of lysosomes in cells was observed by two photon laser scanning microscopy. Before irradiation, DSPE@M-SiPc was highly clustered in the lysosomes. After irradiation, the red fluorescence of DSPE@M-SiPc in lysosomes dispersed, and the cells shrank and blistered (Figure 3D), indicating that the lysosomal tissue in the cells was damaged after PDT. We speculate that the destruction of lysosomes by PDT seriously affected cell function and ultimately led to cell apoptosis. Therefore, DSPE@M-SiPc is expected to be a promising photosensitizer for photodynamic therapy.

## 4 Conculsion

A novel morpholinyl silicon phthalocyanine (M-SiPc) and its water-soluble nanoparticle form (DSPE@M-SiPc) were prepared and characterized. They possessed multifunctional abilities, including lysosome targeting capability, two-photon bio-imaging, and *in vitro* photodynamic therapy. Based on the low toxicity, water solubility, and two photon fluorescence of DSPE@M-SiPc, we concluded that it can be used as a novel and sensitive two photon probe for labeling lysosomes. Moreover, DSPE@M-SiPc can produce ROS in living cells and exhibited excellent phototoxicity effects against MCF-7 cells, leading to cell apoptosis via the destruction of the function of lysosomes. These results clearly highlighted the potential application of DSPE@M-SiPc as a nano-photosensitizer candidate for bioimaging-guided *in vitro* PDT of cancers, as well as a sensitive probe for labeling lysosomes.

## Data Availability

The original contributions presented in the study are included in the article/Supplementary Material, further inquiries can be directed to the corresponding authors.
