# Supplementary material for: Morpholinyl silicon phthalocyanine nanoparticles with lysosome cell death and two-photon imaging functions for in vitro photodynamic therapy of cancer cells
Source: Front Bioeng Biotechnol. 2023 May 5;11:1181448. doi: 10.3389/fbioe.2023.1181448 (PMC10196173; doi:10.3389/fbioe.2023.1181448)
Supplement: Supplementary file 1 [file DataSheet1.doc]

A morpholinyl silicon phthalocyanine nanoparticles with lysosome cell death and two-photon imaging functions for in vitro photodynamic therapy of cancer cells

1. Photophysical propertie

The determination conditions of UV-Vis Spectrum: 25 ℃, scanning wavelength is 200-800 nm.

Fluorescence spectrum determination conditions: the excitation spectrum of M-SiPc was determined at 25 ℃, and the excitation wavelength with the strongest fluorescence intensity of M-SiPc was obtained from the excitation spectrum. The fluorescence emission spectrum of M-SiPc and DSPE@M-SiPc was measured by using this excitation wavelength.

The entrapment efficiency and drug loading efficiency of M-SiPc in DSPE@M-SiPc can be calculated according to formulas S1 and S2.

$EE\%=\frac{M}{M_{0}}\times100\%$(Eq. S1)


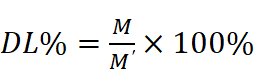
 (Eq. S2)

Where M is the mass of M-SiPc in DSPE-PEG_2000_; M_0_ is the mass of M-SiPc added before loading; M' is the mass of amphiphilic copolymer DSPE-PEG_2000_ (in this paper, all are 1mg).

The fluorescence quantum yield: n-ZnPc (DMF, Ф_F_=0.28) as reference [S1]. According to formula S3, the fluorescence quantum yields of M-SiPc and DSPE@M-SiPc were calculated.


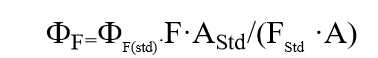
(Eq. S3)

Note: A_Std_ is the absorbance at the excitation wavelength of standard n-ZnPc; A is the absorbance of M-SiPc/ DSPE@M-SiPc at the excitation wavelength; F_Std_ is the integral area of standard n-ZnPc fluorescence spectrum; F is the integral area of the fluorescence spectra of M-SiPc/ DSPE@M-SiPc to be measured.

Quantum yield of singlet oxygen: 25 ℃, irradiated by 671 nm laser in the condition of continuous oxygen supply by using chemical trapping method, 1,3-diphenylisobenzofuran (DPBF) and 9,10-anthracene-bis (methylene) malonic acid (ABDA) were used as trapping agents for singlet oxygen in DMF and H_2_O respectively. The photodegradation of M-SiPc at DPBF (414nm) and ABDA (378nm) was determined by UV-Vis. The singlet oxygen quantum yield of M-SiPc/ DSPE@M-SiPc were calculated by n-ZnPc (DMF, Φ_∆_^ref^= 0.56) as the standard [S2-3]. K is the photobleaching constant of DPBF/ABDA. K^ref^ is the photobleaching constant of DPBF/ABDA in n-ZnPc. I_a_ is the optical absorption rate of the M-SiPc/ DSPE@M-SiPc under laser excitation; I_a_^ref^ is the optical absorption rate of n-ZnPc under laser excitation.


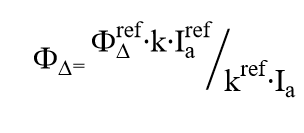
(Eq. S4)


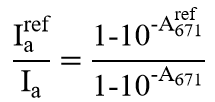
(Eq. S5)

Fluorescence decay curve: at 25 ℃, the fluorescence decay curve of M-SiPc and DSPE@M-SiPc were measured at the maximum emission wavelength by 670 nm laser excitation, and the fluorescence lifetimes of M-SiPc and DSPE@M-SiPc were fitted according to formula S6 [S4].

Fit=A+B_1_e^-t/τ1^+B_2_e^-t/τ2^+B_3_e^-t/τ3^ (Eq. S6)

1. Characterization


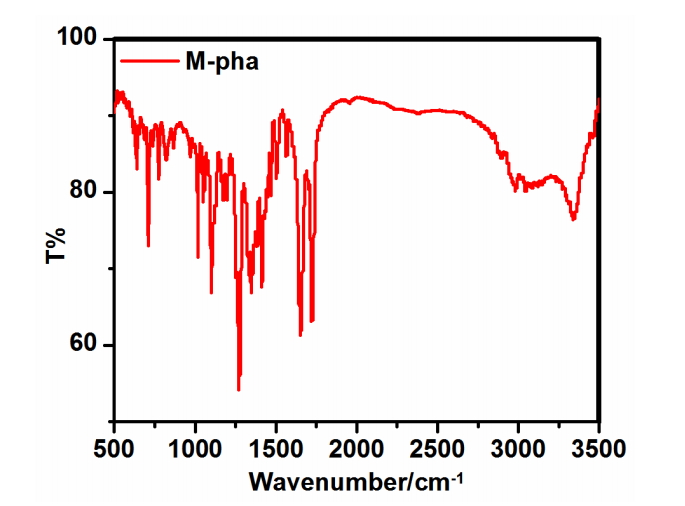


**Fig.S1** The FT-IR spectrum of M-pha


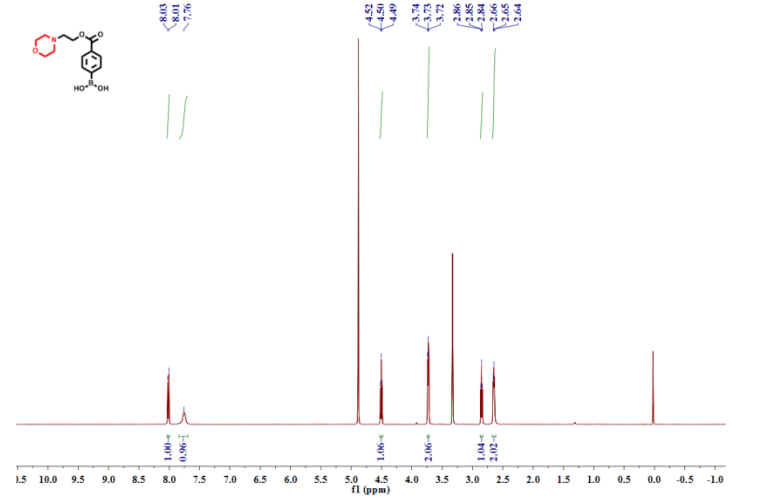


**Fig.S2** The ^1^H NMR spectrum of M-pha (400Hz, CH_3_OD)


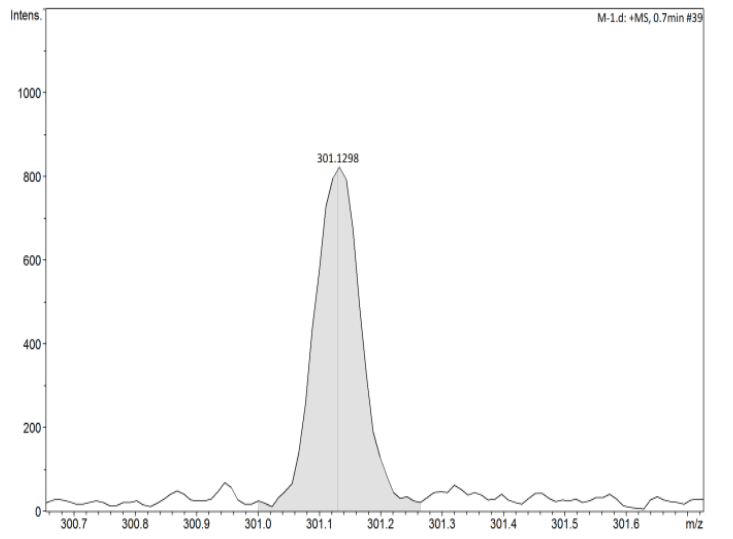


**Fig.S3** The ESI-MS spectrum of M-pha


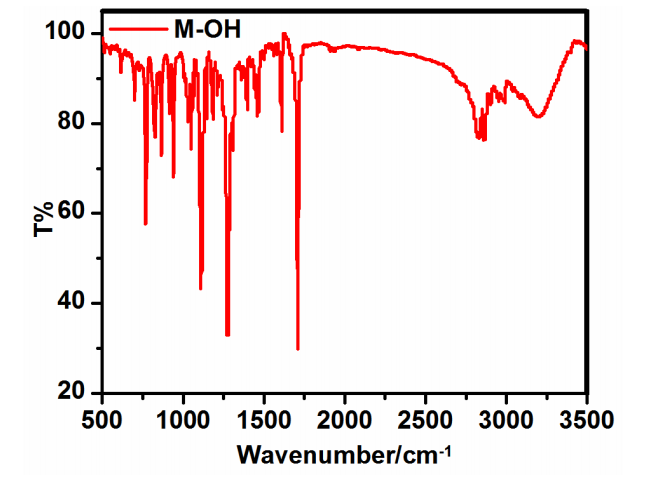


**Fig.S4** The FT-IR spectrum of M-OH


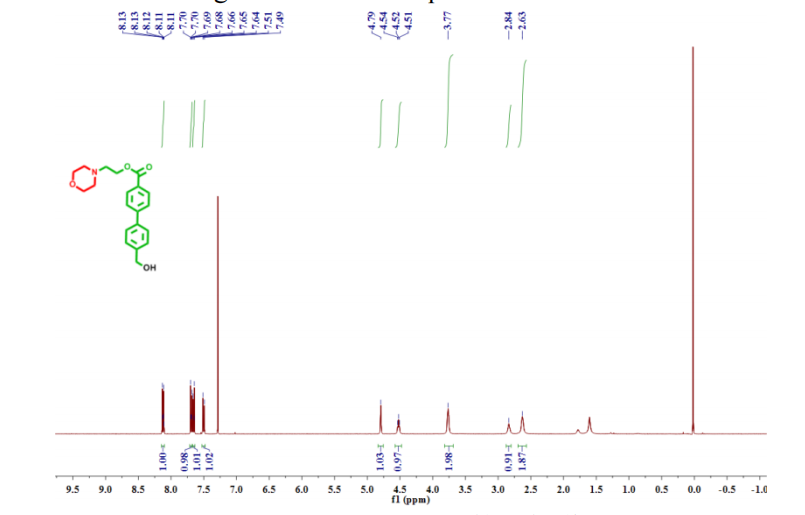


**Fig.S5** The ^1^H NMR spectrum of M-OH (400 MHz, CDCl_3_)


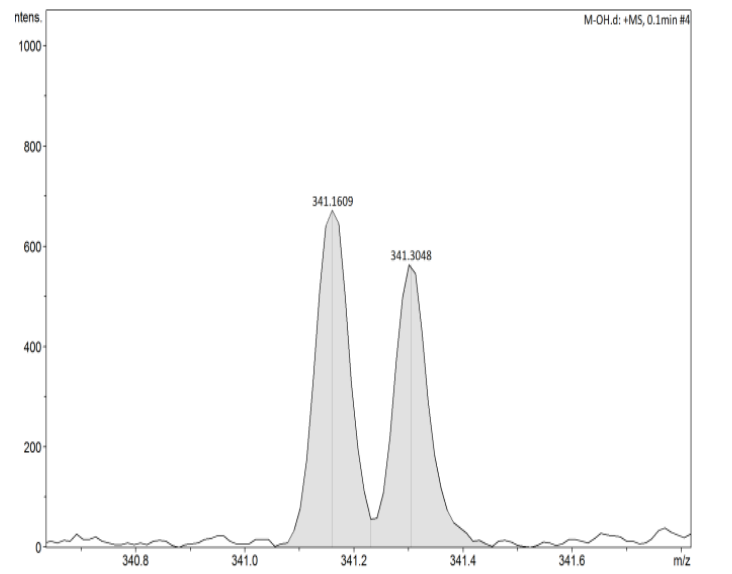


**Fig.S6** The ESI-MS spectrum of M-OH


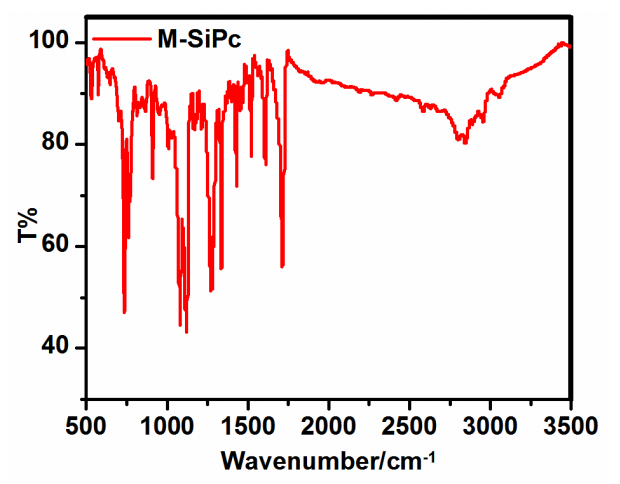


**Fig.S7** The FT-IR spectrum of M-SiPc


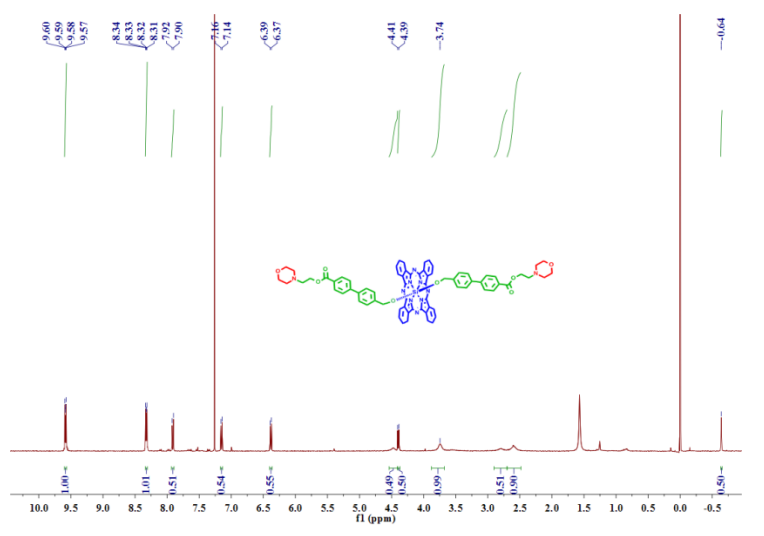


**Fig.S8** The ^1^H NMR spectrum of M-SiPc (400 MHz, CDCl_3_)


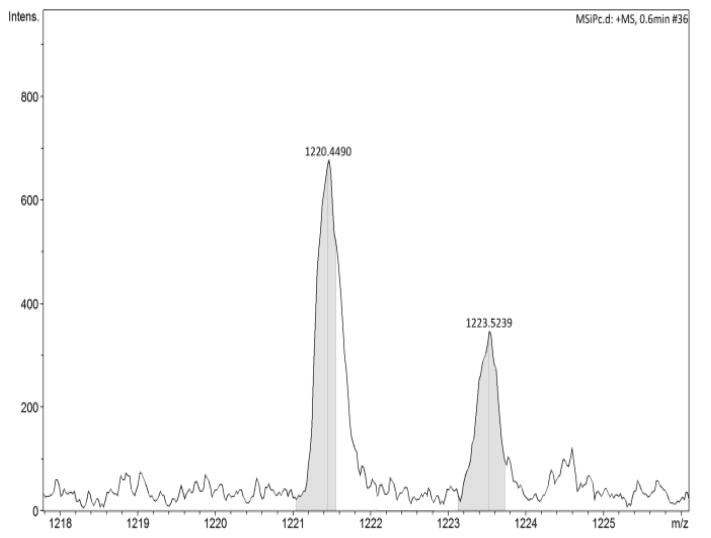


**Fig.S9** The ESI-MS spectrum of M-SiPc

**Fig.S10** UV-vis spectra of DPBF in the present of M-SiPc under irradiation of 670 nm laser (40 mW/cm^2^)

**References**

[S1] Fu J, Li X-Y, Ng D K P, et al. Encapsulation of Phthalocyanines in Biodegradable Poly (sebacic anhydride) Nanoparticles[J]. Langmuir, 2002, 18(10): 3843-3847.

[S2] Chen X, Wu S, Ma D, et al. A polyfluoroalkyl substituted phthalocyanine based supramolecular light switch for photothermal and photodynamic antibacterial activity against Escherichia coli [J]. Chem Commun (Camb), 2018, 54(94): 13279-13282.

[S3] Gorduk S. Octa-substituted metallophthalocyanines bearing (2, 3-dihydrobenzo-1, 4-benzodioxin-2-yl) methoxy and chloro groups: Synthesis, characterization and photophysicochemical studies [J]. Journal of Porphyrins and Phthalocyanines, 2020, 24(04): 548-562.

[S4] Guo Q, Chen L, Pan S, et al. Morpholinyl dendrimer phthalocyanine: synthesis, photophysical properties and photoinduced intramolecular electron transfer [J]. Dalton Trans, 2018, 47(37): 13164-13170.
